# Fractal analysis of dynamic stress myocardial CT perfusion decouples diagnostic accuracy for obstructive coronary artery disease from remote flow

**DOI:** 10.1007/s11604-025-01883-6

**Published:** 2025-10-08

**Authors:** Florian Michallek, Satoshi Nakamura, Masafumi Takafuji, Naoki Nagasawa, Tairo Kurita, Kaoru Dohi, Hideki Ota, Kensuke Nishimiya, Ryo Ogawa, Takehito Shizuka, Hitoshi Nakashima, Naoki Hatori, Yining Wang, Tatsuro Ito, Marc Dewey, Hajime Sakuma, Kakuya Kitagawa

**Affiliations:** 1 Department of Advanced Diagnostic Imaging, Mie University Graduate School of Medicine, 2-174, Edobashi, Tsu, Mie 514-8507 Japan; 2 Department of Radiology, Charité – Universitätsmedizin Berlin, corporate member of Freie Universität Berlin, Humboldt-Universität zu Berlin, and Berlin Institute of Health, Berlin, Germany; 3 Department of Radiology, Mie University Graduate School of Medicine, Tsu, Japan; 4 Department of Radiological Technology, Faculty of Health Science, Suzuka University of Medical Science, Suzuka, Japan; 5 Department of Cardiology and Nephrology, Mie University Graduate School of Medicine, Tsu, Japan; 6 Department of Diagnostic Radiology, Tohoku University Graduate School of Medicine, Sendai, Japan; 7 Department of Cardiology, Tohoku University Graduate School of Medicine, Sendai, Japan; 8 Department of Radiology, Ehime University Graduate School of Medicine, Matsuyama, Japan; 9 Department of Cardiology, National Hospital Organization Takasaki General Medical Center, Takasaki, Japan; 10 Department of Cardiovascular Medicine, National Hospital Organization Kagoshima Medical Center, Kagoshima, Japan; 11 Department of Radiology, Peking Union Medical College Hospital, Chinese Academy of Medical Sciences and Peking Union Medical College, Beijing, China; 12 Department of Cardiovascular Medicine, Kobe University Graduate School of Medicine, Kobe, Japan; 13 DZHK (German Center for Cardiovascular Research), partner site Berlin, Berlin, Germany; 14 Regional Co-creation Deployment Center, Mie Regional Plan Co-creation Organization, Tsu, Japan

**Keywords:** Coronary artery disease, Microvascular ischemia, Myocardial perfusion imaging, Fractals, Computed tomography

## Abstract

**Purpose:**

In patients with reduced remote flow, dynamic stress CT perfusion imaging (CTP) has limited utility for detecting obstructive coronary artery disease (CAD). We compared fractal analysis, a descriptor of macro- and microvascular ischemia patterns, with relative myocardial blood flow (MBF) for detecting obstructive CAD stratified by remote flow.

**Materials and methods:**

This secondary analysis of the prospective multi-center AMPLIFiED trial included patients who underwent invasive coronary angiography (ICA) with fractional flow reserve (FFR) and dynamic stress CTP. Obstructive CAD was defined invasively (stenosis ≥ 90% or FFR < 0.8). We assessed diagnostic accuracy of fractal analysis and relative MBF in patient groups subdivided into high, intermediate, and low remote flow.

**Results:**

In 148 patients (30% female; 416 vessels), obstructive CAD was present in 71/148 patients (48%), while signs of microvascular ischemia were found in 26/148 patients (prevalence in high, intermediate, low remote flow: 9%, 37%, 70%). Fractal analysis outperformed relative MBF in detecting obstructive CAD (patient-based sensitivity and specificity: 94% and 92% versus 80% and 64%, *p* < 0.005). In vessel-based subgroup analysis, both methods performed comparably in high remote flow (area under the receiver-operating curve [AUC]: 0.94 versus 0.88, *p* = 0.45), while fractal analysis outperformed relative MBF in intermediate and low remote flow (AUC: 0.93 versus 0.77, *p* = 0.004; 0.92 versus 0.6, *p* < 0.001).

**Conclusion:**

Fractal analysis eliminates reliance on remote flow for CTP imaging, which improves diagnostic accuracy for obstructive CAD particularly in patients with reduced remote flow or microvascular ischemia.

**Trial registration:**

This study reports about data from the prospective, multi-center AMPLIFiED trial (UMIN000016353).

## Introduction

Dynamic stress computed tomography myocardial perfusion imaging (CTP) has emerged as a promising tool for the non-invasive evaluation of coronary artery disease (CAD), providing functional information to overcome the limited specificity of coronary CT angiography (CTA) in assessing the hemodynamic significance of coronary lesions [1–4]. By providing quantitative assessment of myocardial blood flow (MBF) during pharmacologically induced stress, CTP offers insights into the myocardial relevance of coronary stenoses, potentially reducing the need for evaluation by invasive coronary angiography (ICA) [5]. However, the interpretation of CTP can be challenging, particularly when conclusive obstructive disease is absent, and microvascular ischemia contributes to a reduction in MBF. In such cases, microvascular ischemia represents a confounder which limits the diagnostic performance of CTP for both absolute and relative MBF measurements [6]. While relative MBF, which normalizes MBF in ischemic myocardial regions to that of remote, non-ischemic regions, has demonstrated improved performance compared to absolute MBF in certain settings, its utility may be limited in patients with globally reduced myocardial perfusion, e.g., due to microvascular ischemia. In such cases, normalization to remote myocardium might obscure the presence of hemodynamically relevant epicardial coronary stenosis, leading to underestimation of disease severity.

The relevance of the microcirculation is increasingly recognized as a contributor to ischemia in patients with and without obstructive CAD, especially in women [7–9]. Several microvascular pathomechanisms have been identified, which have the potential to limit vasodilator capacity and, hence, might reduce MBF during stress testing [10]. This poses a diagnostic challenge, as reduced MBF can be caused by epicardial stenosis and microvascular ischemia, with only the former being potentially suitable for invasive revascularization strategies. As such, there is a need for clinically available, non-invasive assessment methods to distinguish between these two pathophysiological entities.

Fractal analysis offers a promising approach to enhance the diagnostic performance of CTP by quantitatively assessing the geometrical complexity of myocardial perfusion [11–13]. Fractals, which describe complex, self-similar patterns across multiple spatial scales, are used in this context to analyze the distribution of perfusion within the myocardium. The fractal dimension (FD) provides a mathematical representation of perfusion complexity, which reflects the integrity of both epicardial and microvascular circulation. By characterizing the perfusion pattern, fractal analysis has the potential to differentiate between obstructive CAD and microvascular disease, which might be helpful particularly in patients with reduced overall MBF.

In this study, we hypothesize that fractal analysis of dynamic stress CTP improves the diagnostic accuracy for detecting obstructive CAD, particularly in patients with low and intermediate absolute remote flow, where microvascular ischemia is more likely to be present. We further hypothesize that fractal analysis outperforms relative MBF due to eliminating microvascular ischemia as confounding factor. To test these hypotheses, we performed a comprehensive analysis of CTP data from a previous multi-center trial, comparing fractal analysis to relative MBF for diagnosing obstructive CAD and examining the impact of remote MBF on diagnostic performance.

## Materials and methods

### Study population

This study utilized data from the prospective, observational, multi-center AMPLIFiED trial (“Assessment of myocardial perfusion linked to infarction and fibrosis explored with a dual-source CT”, UMIN-Clinical Trial Registry number: UMIN000016353) conducted at seven centers in Japan (six centers) and China (one center), enrolling patients with a clinical indication for invasive coronary angiography (ICA) due to suspected or known coronary artery disease (CAD) [2]. Myocardial segments showing delayed enhancement on CT were excluded from perfusion analysis to avoid confounding by chronic myocardial infarction or fibrosis alongside associated tissue remodeling, which includes re-establishment of microvasculature via collateral circulation and alterations in the extracellular space. Vessels were excluded if all pertaining segments showed delayed enhancement. Vessels with both infarcted and non-infarcted segments were included if the non-infarcted segments could be analyzed (e.g., in isolated branch infarction). Patients were excluded at the per-patient level if all vessels were excluded or if infarcted vessels coexisted with otherwise normal vessel territories, to avoid misclassification as normal perfusion. Patients with prior coronary artery bypass grafting (CABG) or contraindications to cardiac CT or adenosine stress testing were also excluded. This study was performed in accordance with the Declaration of Helsinki. The central Institutional Review Board (the Clinical Research Ethics Review Committee of Mie University Hospital) and the local Institutional Review Boards of participating centers approved the research protocol.

### CT imaging protocol

All patients underwent comprehensive cardiac CT imaging, including calcium scoring, dynamic stress CTP, CT angiography (CTA), and delayed enhancement imaging. Scans were performed using second or third-generation dual-source CT scanners (Somatom Definition Flash or Somatom Force, Siemens Healthineers), depending on the center. Pharmacological stress was induced by administration of adenosine triphosphate (ATP) over at least 3 min at a rate of 160 μg/kg/min. Dynamic CTP acquisition was acquired in end-systolic phase (250 ms after the R wave) for 30 s immediately after the administration of contrast agent (40 mL iopamidol at 5 mL/s) followed by a saline flush. Acquisition was performed using ECG-triggered axial scanning in shuttle mode (two table positions alternating every heartbeat), with parameters as follows using second or third-generation scanners, respectively: z-axis coverage 73 mm or 105 mm, collimation 32 × 1.2 or 48 × 1.2 mm, rotation time 0.28 or 0.25 s, tube voltage 80 or 70 kV. Tube current was modulated by automatic exposure control, using a quality reference of 350 at 120 kV or 300 mAs/rotation at 80 kV. CT angiography (CTA) was performed 10 min after dynamic stress CTP using a standard prospective acquisition mode after administration of sublingual nitrates for vasodilation and contrast agent (iopamidol at 26 mg iodine/kg/s over 8 s). Where necessary, heart rate was controlled prior to coronary CTA by intravenous beta-blocker injection. Delayed enhancement CT was acquired with a 5-min delay after the CTA contrast agent injection. Details on the imaging protocols are given in [2].

### Image analysis

All image analysis steps in this study were performed using custom software developed by the first author. The principal steps of the image analysis procedure are summarized here, and full methodological details can be found in [12]: dynamic CTP datasets were first registered using a principal component analysis-based, non-rigid registration scheme, which allows simultaneous four-dimensional image registration and is robust against the time-attenuation effects of inflowing contrast agent [12, 14].

Noise was standardized through a two-step denoising approach: a median filter (3 × 3 × 3 pixels), followed by a bilateral filter with a spatial (domain) standard deviation *σ*_domain_ = 2 and an individualized intensity range standard deviation *σ*_range_ = 1.96 * standard deviation of unenhanced left ventricular myocardium. This approach allows to adapt the strength of the denoising to the individual noise level in each dataset. Subsequently, Hounsfield units were converted to absolute contrast agent concentrations [15].

Myocardial segmentation followed the 17-segment model of the American Heart Association (AHA). ROIs were placed manually within each segment, maintaining at least 1 mm distance from endocardial and epicardial borders to avoid partial volume effects. In cases of partial ischemic defects, ROIs were confined to the defect sub-regions. From those ROIs, the mean values of the fractal dimension maps and MBF maps were extracted and subjected to statistical analysis.

The entire image analysis procedure took about 20 min per patient and comprised the above steps as well as the calculation times for fractal dimension maps (about 2.5 min) and MBF maps (about 1 min) on a standard clinical workstation.

### Fractal analysis of myocardial perfusion

Fractal analysis provides the fractal dimension as a quantitative measure of the spatial and temporal complexity of myocardial perfusion patterns, reflecting the hierarchical and scale-invariant organization of the coronary vasculature. Fractal dimension (FD) was calculated using the blanket algorithm applied to the 4D perfusion dataset as previously established and validated [11–13], which simultaneously integrates spatial and temporal texture dynamics without requiring image binarization. The blanket algorithm quantifies changes in image texture across spatial scales by measuring the decline in local intensity variation with increasing scale, thereby capturing the fractal geometric complexity of perfusion patterns. The upper blanket *u*_ε_ and lower blanket *b*_ε_ are defined as:

1 $$u_{\varepsilon } \left( {i,j,k,t} \right) = \max \left\{ {u_{\varepsilon - 1} \left( {i,j,k,t} \right) + 1,\mathop {\max }\limits_{{\left| {\left( {m,n,o,s} \right) - \left( {i,j,k,t} \right)} \right| \le 1}} u_{\varepsilon - 1} \left( {m,n,o,s} \right)} \right\}$$

2 $$b_{\varepsilon } \left( {i,j,k,t} \right) = \min \left\{ {b_{\varepsilon - 1} \left( {i,j,k,t} \right) - 1,\mathop {\min }\limits_{{\left| {\left( {m,n,o,s} \right) - \left( {i,j,k,t} \right)} \right| \le 1}} b_{\varepsilon - 1} \left( {m,n,o,s} \right)} \right\}$$

The variables are defined as follows: ε represents scale (implemented as iteration counter), i, j, k, t, m, n, o, s represent four-dimensional pixel coordinates (three spatial dimensions, one temporal dimension). Together, these blankets define the hypervolume V(ε), which is quantified at each iteration as:

3 $$V\left( \varepsilon \right) = \frac{{\mathop \sum \nolimits_{i,j,k,t} \left( {u_{\varepsilon } \left( {i,j,k,t} \right) - b_{\varepsilon } \left( {i,j,k,t} \right)} \right)}}{2\varepsilon }$$

Since the myocardial texture has fractal properties due to its underlying biological origin (i.e., perfusion), the bi-logarithmic diagram of log *V*(*ε*) ~ log ε yields a straight line, the slope of which is inserted into the following equation for calculating the fractal dimension FD:

4 $$\mathrm{FD}=4-\mathrm{slope}$$

The algorithm operates in local kernels with a diameter of three in each of the four dimensions and, hence, yields maps of the local FD, which can be displayed as an overlay on top of the myocardium and can be assessed in an analogous manner to local MBF maps. Those myocardial FD maps represent the spatio-temporal complexity of contrast agent distribution (i.e., the observable perfusion pattern). In the context of ischemia imaging, higher FD values indicate a physiological perfusion pattern due to the fractally complex underlying vascular architecture. In contrast, lower FD values reflect a loss in perfusion complexity found in ischemia—with a moderate loss in microvascular ischemia and a severe loss in obstructive CAD.

Using the AHA 17-segment model, FD was obtained for each myocardial segment. Cut-off FD values for obstructive CAD (FD ≤ 4.31), microvascular ischemia (4.31 < FD ≤ 4.41), and normal perfusion (FD > 4.41) have been derived and independently validated in previous studies [11–13].

### Myocardial blood flow measurements

We calculated MBF for each myocardial segment according to the 17-segment model of the American Heart Association using the previously established maximum-upslope method for dynamic stress CTP data [2, 16]. Segment-wise MBF values were normalized to blood flow in remote myocardium (“remote flow”), defined as the AHA segment with the highest MBF. These normalized MBF values were expressed as “relative MBF” and used for comparison against fractal analysis. Based on the absolute value of remote flow, patients were stratified into three subgroups, i.e., high, intermediate, and low remote flow, using tertiles of the patient-based remote flow distribution.

### Invasive coronary angiography and fractional flow reserve

Invasive coronary angiography was performed within 60 days of the cardiac CT using standard techniques, with intraprocedural visual assessment of coronary stenosis severity by experienced operators. Fractional flow reserve (FFR) measurements were obtained in coronary vessels with stenosis > 25% and < 90% on ICA. Stenoses with FFR < 0.8 were considered hemodynamically significant.

### Statistical analysis

Obstructive CAD was defined as a stenosis ≥ 90% on ICA or FFR < 0.8 on the vessel level. On the patient level, obstructive CAD was diagnosed if at least one coronary artery met the criteria for obstructive CAD. Microvascular ischemia was defined as evidence of ischemia on the patient level in the absence of corresponding obstructive CAD as defined above. To assess the overall burden of coronary lesions per patient, we calculated segment-stenosis score and segment-involvement score [17].

Cut-off values for FD have been derived and independently validated in previous studies [11–13] and were adhered to in this study. Cut-off values for relative MBF were derived from the Youden index in receiver-operating characteristic (ROC) analysis on the vessel level. Diagnostic performance of fractal analysis and relative MBF for detecting obstructive CAD was assessed on both the vessel and patient levels by contingency tables and ROC analysis with calculation of areas under the ROC curves (AUC). Sensitivity and specificity of each method were compared by McNemar’s Chi-square test, and the DeLong test was used to compare AUC values. Subgroup analysis was performed based on absolute remote flow: patients were stratified into high, intermediate, and low remote flow groups based on tertiles of the per-patient remote flow distribution, and the diagnostic performance of each method was compared within these groups on the patient and vessel levels. Cohen’s κ was used to assess inter-reader variability in a subset of 30 patients. We excluded patients from analysis with extensive infarction. Vessels were excluded from analysis if they were hypoplastic or if FFR data was missing, despite the presence of intermediate stenosis. Statistical significance was defined at two-sided *p* ≤ 0.05. All statistical analyses were performed using R (version 4.4.1, R Core Team, Vienna, Austria). The STARD guidelines have been adhered to.

## Results

### Study population

**Fig. 1:**
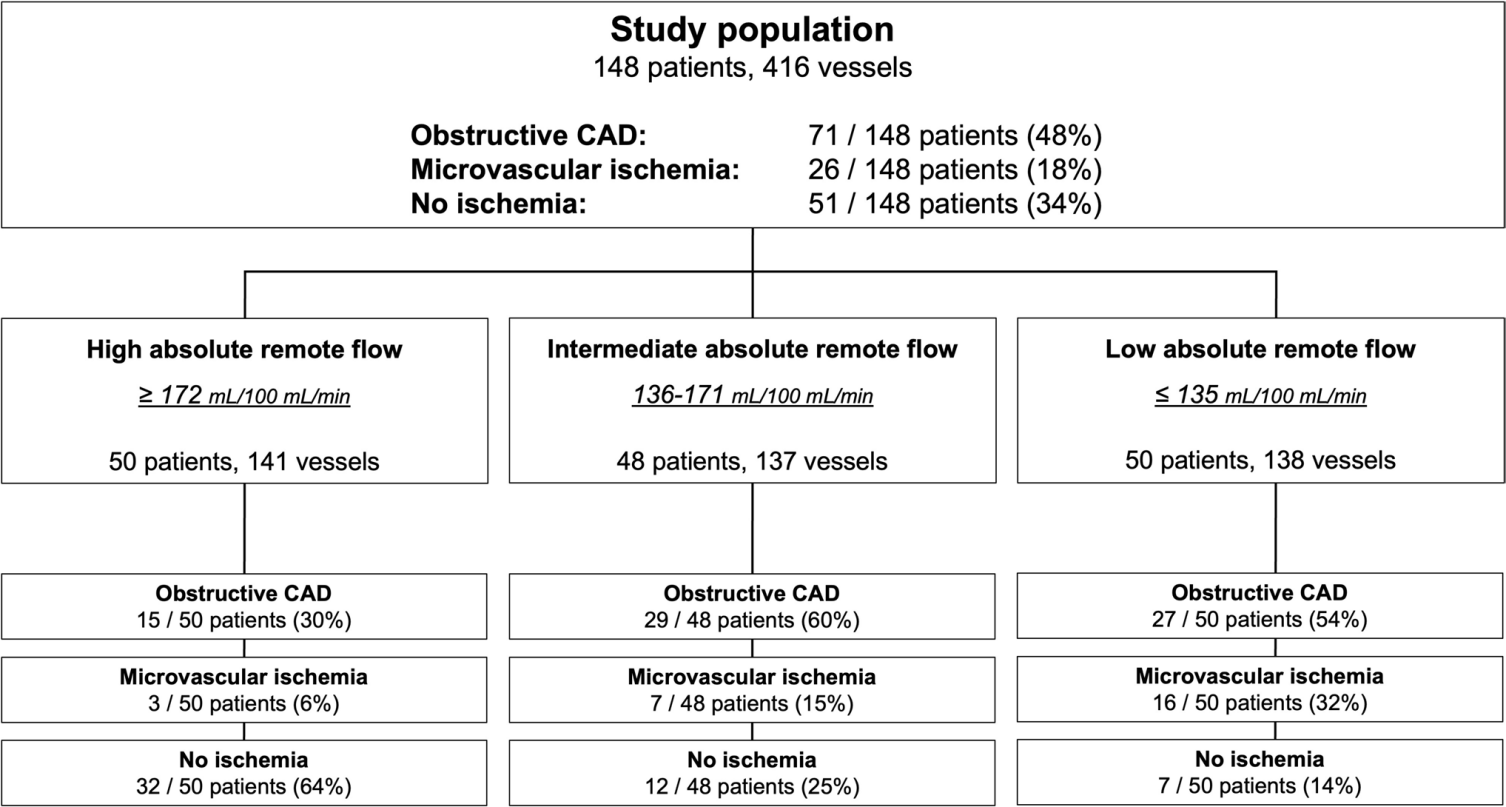
Study population stratified by remote flow groups. *CAD* coronary artery disease

A total of 148 patients (median age 67 ± 13 years, 30% female) with 416 coronary vessels were included in the analysis (Fig. 1). Nine patients were excluded in whom perfusion defects entirely coincided with delayed enhancement as specified in the exclusion criteria. Of those nine patients, four patients had no obstructive disease, two patients had one-vessel disease, one patient had two-vessel disease, and two patients had three-vessel disease. Obstructive CAD was present in 71/148 patients (48%) and, respectively, in 86/416 vessels (21%). From the 71 patients with obstructive disease, 45 patients had one-vessel disease, 21 patients had two-vessel disease, and five patients had three-vessel disease. Median segment-stenosis score was 7 (interquartile interval: 3–12) and median segment-involvement score was 6 (interquartile interval: 2–9). Baseline demographic and clinical characteristics are shown in Table 1. While cut-off values for FD were obtained previously [11–13], the optimal cut-off value for relative MBF was found at ≤ 0.674 to predict obstructive CAD. Inter-reader variability of fractal analysis was high (Cohen’s *κ* = 0.94, CI: 0.92–0.96). The comprehensive imaging protocol resulted in a mean effective radiation dose of 12.6 ± 4.1 mSv, including 4.9 ± 1.2 mSv specifically for dynamic stress CTP with a conversion coefficient *k* = 0.014 (Table 1).

**Table 1:** Patient characteristics

	All patients	High remote flow	Intermediate remote flow	Low remote flow	*p*
*n*, patients	148	50	48	50	
*n*, vessels	416	138	137	141	
Remote flow for subgroup assignment (mL/100 mL/min)	51–253	≥ 172	171–136	≤ 135	
Age, years (median ± IQR)	67 ± 13	67.5 ± 12.8	70.5 ± 12.3	64 ± 15	**0.03**
Female, *n* (%)	45 (30)	20 (40)	13 (27)	12 (24)	0.19
Risk factors					
Body mass index (mean ± SD)	24.5 ± 3.9	24.2 ± 3.6	24 ± 3.4	25.4 ± 4.6	0.13
Arterial hypertension, *n* (%)	100 (68)	33 (66)	32 (67)	35 (70)	0.74
Diabetes, *n* (%)	44 (30)	10 (20)	16 (33)	18 (36)	0.13
Dyslipidemia, *n* (%)	81 (55)	28 (56)	27 (56)	26 (52)	0.95
Smoking, *n* (%)	62 (42)	21 (42)	22 (46)	19 (38)	0.77
Family history of CAD, *n* (%)	16 (11)	8 (16)	4 (8)	4 (8)	0.39
Creatinine, mg/dL (mean ± SD)	0.8 ± 0.19	0.74 ± 0.15	0.82 ± 0.18	0.83 ± 0.24	0.09
CT effective radiation dose					
Total*, mSv (mean ± SD; *k* = 0.014)	12.6 ± 4.1	11.3 ± 2.8	12.8 ± 4.6	13.7 ± 4.7	**0.02**
Stress CTP (mean ± SD; *k* = 0.014)	4.9 ± 1.2	4.8 ± 1.1	4.7 ± 1.2	5.1 ± 1.4	0.41

*IQR* interquartile range, *SD* standard deviation, *k* conversion coefficient 0.014

*P* significance level (bold: *p* ≤ 0.05)

^*^Total dose including calcium scan, dynamic stress CT perfusion, CT angiography, CT delayed enhancement

### Patient-level diagnostic accuracy

**Fig. 2:**
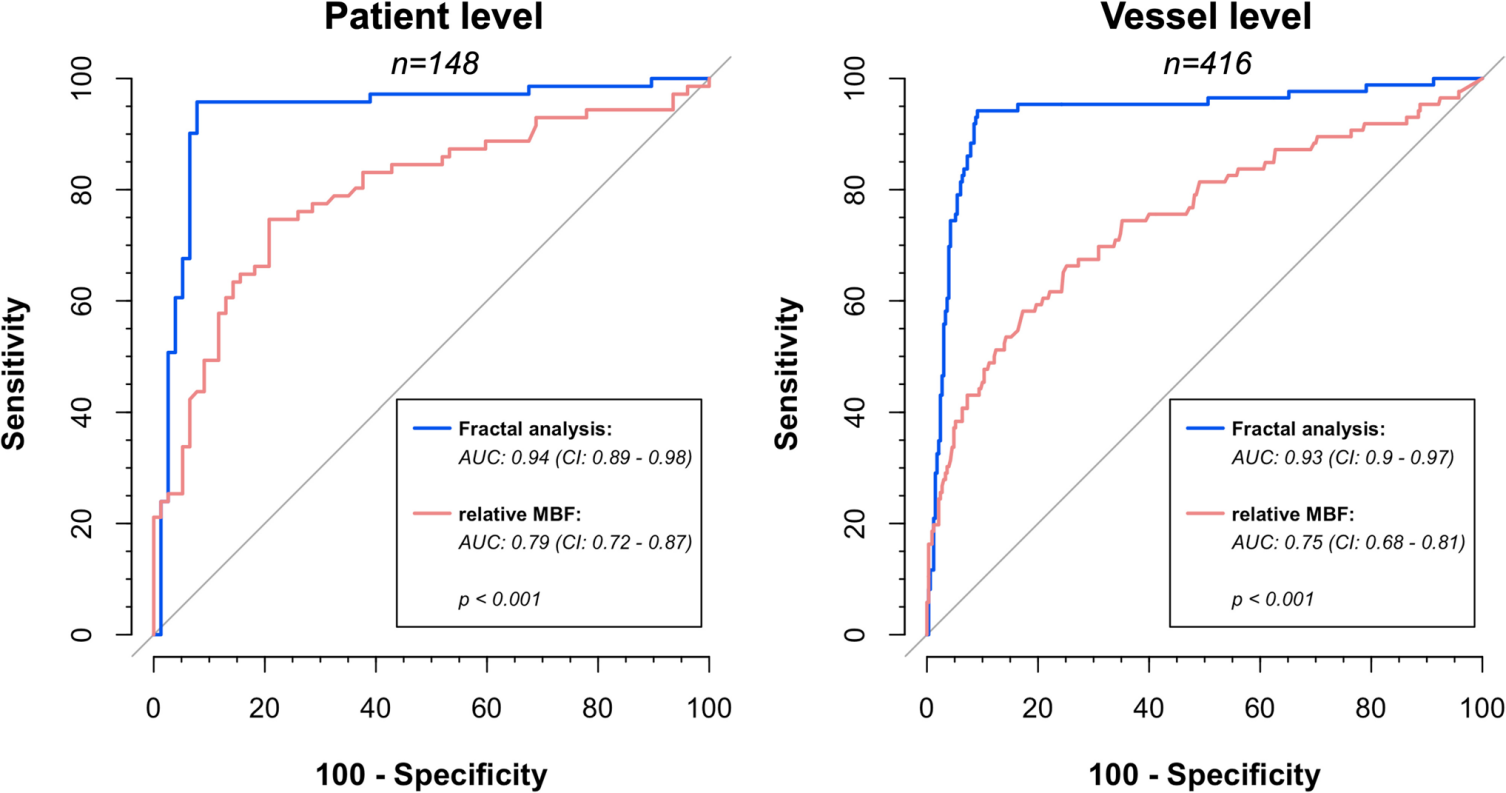
Receiver operating curve (ROC) analysis for diagnosis of obstructive coronary artery disease (CAD) on the patient (left) and vessel level (right). Area under the curve (AUC) was significantly higher for fractal analysis compared to relative myocardial blood flow (MBF) measurements on both levels

At the patient level, fractal analysis demonstrated superior diagnostic performance over relative MBF: Sensitivity and specificity for identifying patients with obstructive CAD were 94% (CI: 86–98%) and 92% (CI: 84–97%) for fractal analysis, compared to 80% (CI: 69–89%) and 64% (CI: 52–74%) for relative MBF (*p* < 0.005). The AUC of fractal analysis was significantly higher compared to relative MBF (AUC 0.94 vs. 0.79, *p* < 0.001), Table 2 and Fig. 2.

**Table 2:** Patient level diagnostic accuracy for obstructive coronary artery disease (CAD)

Patient level	All patients	High remote flow	Intermediate remote flow	Low remote flow
Obstructive CAD, *n* (%)	71/148 (48)	15/50 (30)	29/48 (60)	27/50 (54)
Fractal analysis				
Sensitivity	**94%** (CI: 86–98%) 67/71	87% (CI: 60–98%) 13/15	93% (CI: 77–99%) 27/29	**100%** (CI: 87–100%) 27/27
Specificity	**92%** (CI: 84–97%) 71/77	**100%** (CI: 90–100%) 35/35	**95%** (CI: 74–100%) 18/19	**78%** (CI: 56–93%) 18/23
AUC	**0.94** (CI: 0.89–0.98)	0.95 (CI: 0.84–1)	0.96 (CI: 0.89–1.0)	**0.89** (CI: 0.78–1.0)
Relative MBF				
Sensitivity	**80%** (CI: 69–89%) 57/71	80% (CI: 52–96%) 12/15	90% (CI: 73–98%) 26/29	**70%** (CI: 50–86%) 19/27
Specificity	**64%** (CI: 52–74%) 49/77	**77%** (CI: 60–90%) 27/35	**58%** (CI: 33–80%) 11/19	**48%** (CI: 27–69%) 11/23
AUC	**0.79** (CI: 0.72–0.87)	0.83 (CI: 0.69–0.98)	0.87 (CI: 0.76–0.97)	**0.62** (CI: 0.46–0.78)

High remote flow: ≥ 172 mL/100 mL/min, intermediate remote flow: 136–171 mL/100 mL/min, low remote flow: ≤ 135 mL/100 mL/min

*MBF* myocardial blood flow, *CI* 95% confidence interval

Bold font face indicates significant differences (*p* ≤ 0.05) in corresponding sensitivity or specificity (McNemar’s Chi-square test), or area under the receiver-operating curves (AUC; DeLong test)

### Vessel-level diagnostic accuracy

Also at the vessel level, fractal analysis outperformed relative MBF: Sensitivity and specificity were 93% (CI: 85–97%) and 91% (CI: 87–94%) for fractal analysis, compared to 66% (CI: 55–76%) and 75% (CI: 70–79%) for relative MBF (*p* < 0.001). AUC was 0.93 vs. 0.75 (*p* < 0.001) for fractal analysis and relative MBF, respectively (Table 3 and Fig. 2).

**Table 3:** Vessel level diagnostic accuracy for obstructive coronary artery disease (CAD)

Vessel level	All vessels	High remote flow	Intermediate remote flow	Low remote flow
Obstructive CAD, *n* (%)	86/416 (21)	16/141 (11)	38/137 (28)	32/138 (23)
Fractal analysis				
Sensitivity	**93%** (CI: 85–97%) 80/86	81% (CI: 54–96%) 13/16	**92%** (CI: 79–98%) 35/38	**97%** (CI: 84–100%) 31/32
Specificity	**91%** (CI: 88–94%) 301/330	**87%** (CI: 80–93%) 109/125	**96%** (CI: 90–99%) 95/99	**78%** (CI: 69–86%) 83/106
AUC	**0.93** (CI: 0.9–0.97)	0.94 (CI: 0.84–1.0)	**0.93** (CI:0.87–1.0)	**0.92** (CI: 0.87–0.96)
Relative MBF				
Sensitivity	**66%** (CI: 55–76%) 57/86	81% (CI: 54–96%) 13/16	**71%** (CI: 54–85%) 27/38	**53%** (CI: 35–71%) 17/32
Specificity	**75%** (CI: 70–79%) 247/330	**87%** (CI: 80–93%) 109/125	**71%** (CI: 61–79%) 70/99	**64%** (CI: 54–73%) 68/106
AUC	**0.75** (CI: 0.68–0.81)	0.88 (CI: 0.79–0.98)	**0.77** (CI: 0.68–0.87)	**0.6** (CI: 0.48–0.72)

High remote flow: ≥ 172 mL/100 mL/min, intermediate remote flow: 136–171 mL/100 mL/min, low remote flow: ≤ 135 mL/100 mL/min

*MBF* myocardial blood flow, *CI* 95% confidence interval

Bold font face indicates significant differences (*p* ≤ 0.05) in corresponding sensitivity or specificity (McNemar’s Chi-square test), or area under the receiver-operating curves (AUC; DeLong test)

### Impact of remote flow on diagnostic performance for obstructive CAD

Patients were stratified into three groups based on the tertiles of absolute remote myocardial blood flow within the study group: high absolute remote flow (≥ 172 mL/100 mL/min, 50 patients, 141 vessels), intermediate absolute remote flow (136–171 mL/100 mL/min, 48 patients, 137 vessels), and low absolute remote flow (≤ 135 mL/100 mL/min, 50 patients, 138 vessels), Fig. 1. Prevalence of obstructive CAD was 15/50 (30%) in patients in high remote flow, 29/48 (60%) in patients in intermediate remote flow, and 27/50 (54%) in patients with low remote flow.

**Fig. 3:**
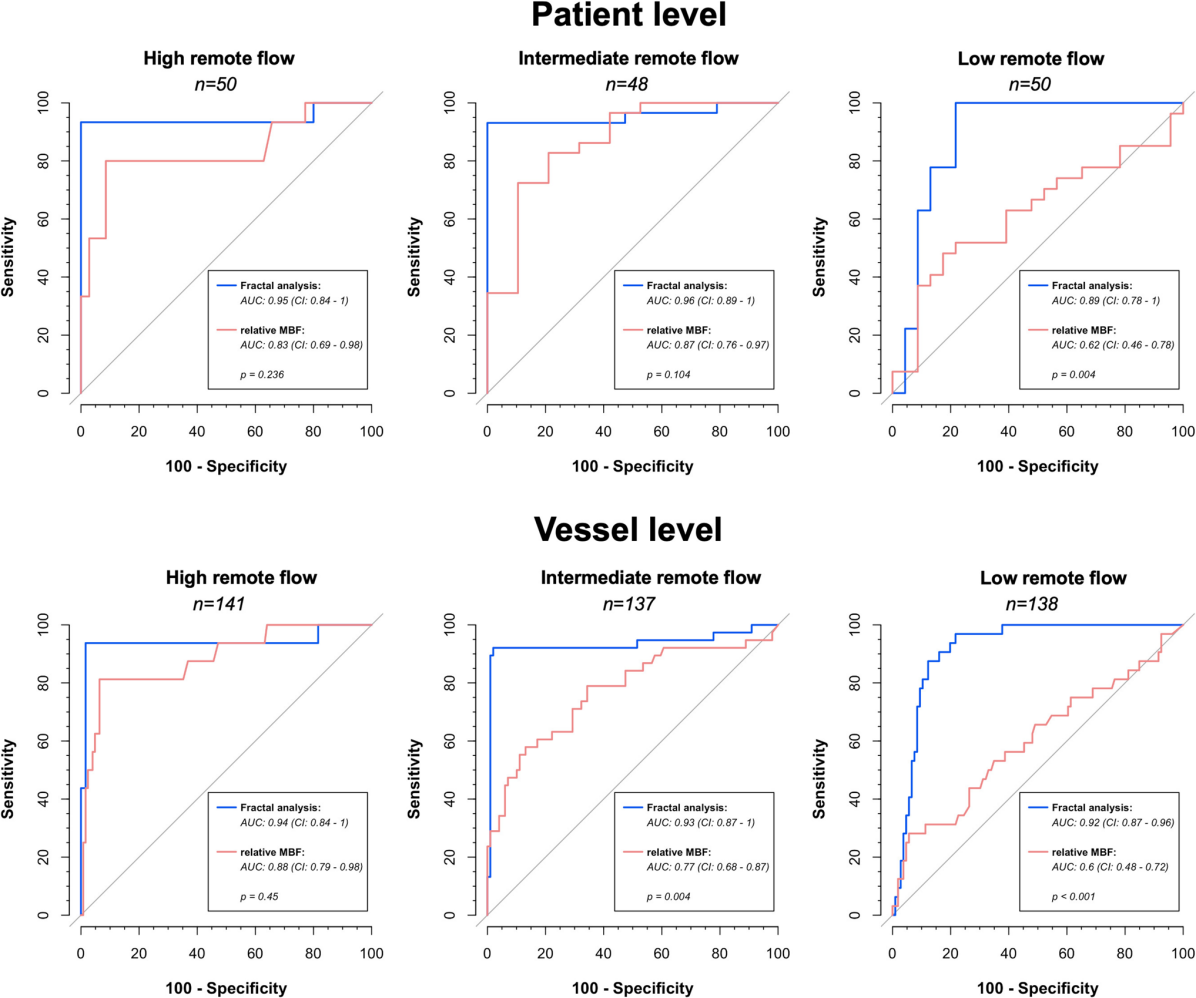
Receiver operating curve (ROC) analysis for diagnosis of obstructive coronary artery disease (CAD) on the patient (top row) and vessel level (bottom row) stratified by remote flow (high remote flow: ≥ 172 mL/100 mL/min, intermediate remote flow: 136–171 mL/100 mL/min, low remote flow: ≤ 135 mL/100 mL/min). Area under the curve (AUC) was significantly higher for fractal analysis compared to relative myocardial blood flow (MBF) measurements in patients with low remote flow as well as vessels with intermediate and low remote flow. In contrast, the other subgroups (patients with high and intermediate remote flow as well as vessels with high remote flow) showed no significant difference albeit AUC being consistently higher for fractal analysis

On the patient level, fractal analysis consistently maintained high diagnostic performance across all remote flow subgroups, while relative MBF only performed well in patients with high and intermediate remote flow but significantly worse in patients with low remote flow (AUC fractal analysis vs. relative MBF: high remote flow 0.95 vs. 0.83, *p* = 0.236; intermediate remote flow 0.96 vs. 0.87, *p* = 0.104; low remote flow 0.89 vs. 0.62, *p* = 0.004), Fig. 3.

On the vessel level, fractal analysis and relative MBF had comparable diagnostic performance only in the high remote flow subgroup (AUC: 0.94 for fractal analysis vs. 0.88 for relative MBF, *p* = 0.45). In contrast, in vessels in the intermediate and low remote flow subgroups, fractal analysis outperformed relative MBF with an AUC of 0.93 vs. 0.77 (*p* = 0.004) in intermediate remote flow and 0.92 vs. 0.6 (*p* < 0.001) in low remote flow.

Interestingly, specificity of fractal analysis was consistently and significantly higher in all patient and vessel subgroups irrespective of remote flow (Tables 2 and 3).

### Presence of microvascular ischemia

**Fig. 4:**
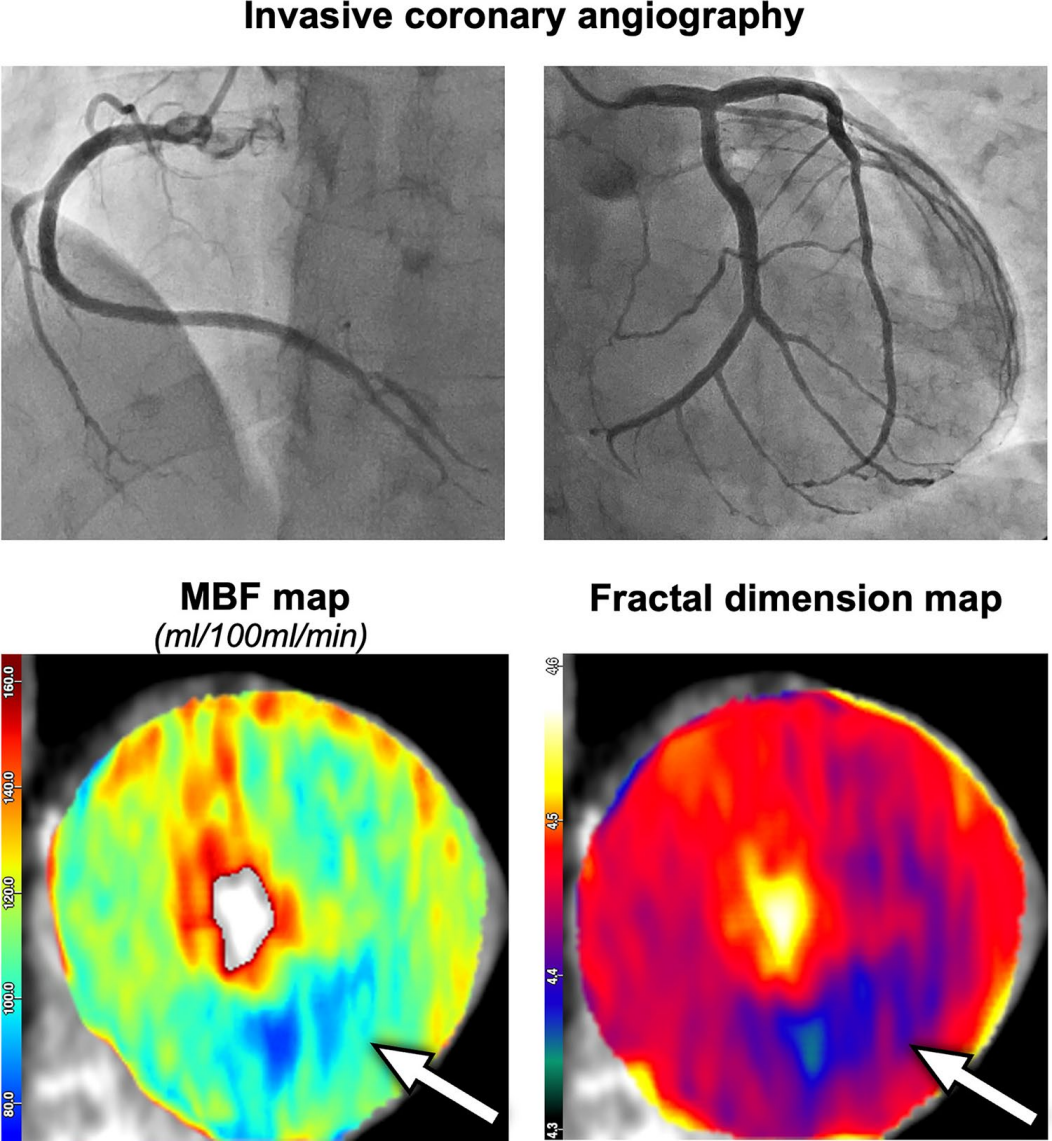
A 63-year-old male patient with a normal invasive angiogram. Remote myocardial blood flow (MBF) was intermediate (149 mL/100 mL/min). A perfusion defect was found inferior in the mid myocardial third as well as in the entire apical third with relative MBF 0.65, i.e., false positive for obstructive coronary artery disease (CAD). Fractal dimension of 4.35 correctly indicated microvascular ischemia

Our study population included 77 patients without obstructive CAD according to the invasive reference standard with FFR, thereof 26 with evidence of microvascular ischemia (representative case in Fig. 4). Their distribution across the remote flow tertiles was as follows: 3/35 (9%) in high remote flow, 7/19 (37%) in intermediate remote flow, and 16/23 (70%) in low remote flow.

We applied the previously established fractal analysis cutoffs at FD = 4.41 and FD = 4.31 [11–13] for differentiating normal perfusion, microvascular ischemia, and obstructive CAD (Table 4). Accordingly, fractal analysis correctly identified 22/26 of the microvascular ischemia patients, while the remaining 4/26 were incorrectly classified as obstructive CAD; none of the 26 microvascular ischemia patients were incorrectly classified as normal perfusion. In patients without ischemia, fractal analysis incorrectly classified 1/51 as microvascular ischemia.

**Table 4:** Identification of microvascular ischemia. Only patients without obstructive coronary artery disease are included in this analysis

Patient level	All patients	High remote flow	Intermediate remote flow	Low remote flow
Microvascular ischemia, *n* (%)	26/77 (34%)	3/35 (9%)	7/19 (37%)	16/23 (70%)
Fractal analysis prediction				
True microvascular	22/26	3/3	6/7	13/16
False obstructive CAD	4/26	0/3	1/7	3/16
False normal	0/26	0/3	0/7	0/16
True normal	50/51	31/32	12/12	7/7
False obstructive CAD	0/51	0/32	0/12	0/7
False microvascular	1/51	1/32	0/12	0/7
Relative MBF prediction				
True microvascular	0/26	0/3	0/7	0/16
False obstructive CAD	15/26	1/3	4/7	10/16
False normal	11/26	2/3	3/7	6/16
True normal	38/51	25/32	8/12	5/7
False obstructive CAD	13/51	7/32	4/12	2/7
False microvascular	0/51	0/32	0/12	0/7

Predictions were based on the vessel level for fractal analysis or relative myocardial blood flow (MBF): patients with at least one-vessel territory classified as obstructive coronary artery disease (CAD) according to the respective method were designated as “obstructive CAD”. Patients with at least one-vessel territory classified as microvascular ischemia without any “obstructive CAD” designation were designated as “microvascular”. The latter, however, applied only to fractal analysis, while a positive relative MBF finding was counted as “obstructive CAD” due to the inability of this method to differentiate between microvascular and obstructive disease. Patients neither classified as “obstructive CAD” nor as “microvascular” were designated as “normal”

In contrast, relative MBF does not allow to differentiate between microvascular ischemia and obstructive CAD; hence, none of the microvascular patients were identified as such. Instead, relative MBF was positive for obstructive CAD in 15/26 microvascular ischemia cases (thereof 10 in the low remote flow subgroup) and normal for the remaining 11/26 patients (thereof 6 in the low remote flow subgroup). In patients without ischemia, relative MBF was positive for obstructive CAD in 13/51 cases (Table 4).

**Fig. 5:**
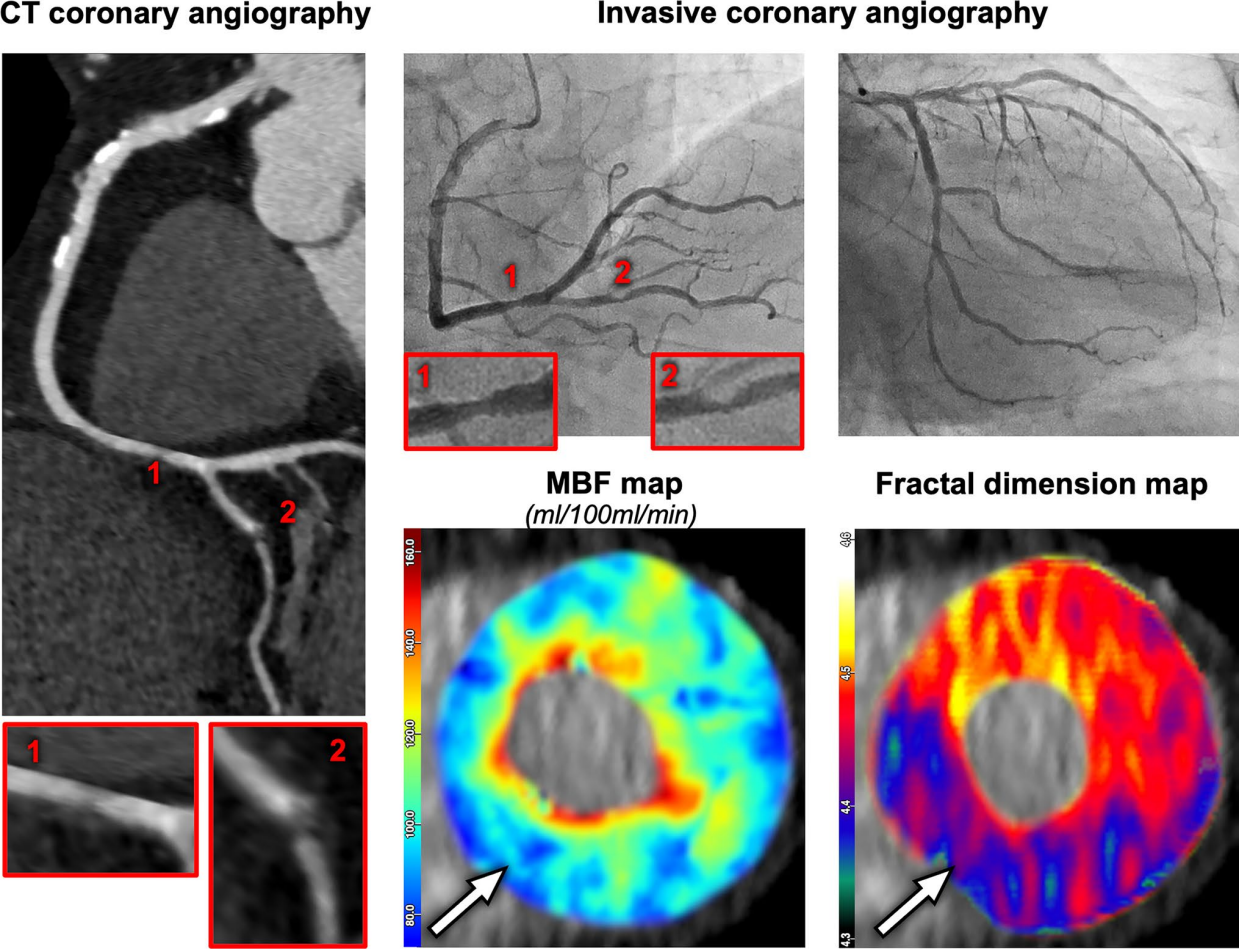
An 84-year-old male patient with 50% stenosis in distal RCA (1) and 75% in PDA (2) with FFR = 0.92, i.e., not hemodynamically relevant, as well as multiple non-significant 25% stenoses in LAD and LCX. Remote myocardial blood flow (MBF) was low (114 mL/100 mL/min). A perfusion defect was found infero-septal to inferior in the mid and apical myocardial thirds with relative MBF 0.62, i.e., false-positive for obstructive coronary artery disease (CAD). Fractal dimension of 4.34 correctly indicated microvascular ischemia

According to ICA, 9/26 microvascular ischemia patients had intermediate stenoses between 50–80% without hemodynamic significance (invasive FFR > 0.8), and fractal analysis correctly classified all of them as microvascular ischemia, as shown as a representative case in Fig. 5.

## Discussion

In this study, we assessed the diagnostic performance of relative MBF and fractal analysis for identifying obstructive CAD across patient subgroups with high, intermediate, and low remote flow, using retrospective data from the multi-center AMPLIFiED trial. Our findings indicate that the diagnostic accuracy of relative MBF for obstructive CAD is limited in patients with reduced remote flow, potentially due to confounding effects of microvascular ischemia. In contrast, fractal analysis, which does not rely on comparison to remote myocardium, maintained high diagnostic accuracy for detecting obstructive CAD even in patients with reduced remote flow. Importantly, the improved specificity of fractal analysis across all remote flow subgroups suggests its effectiveness in distinguishing ischemia due to epicardial stenosis from microvascular ischemia.

A reliable morphological assessment of the coronary arteries is provided by CTA with high sensitivity and negative predictive value regarding obstructive CAD [18]. However, CTA alone is limited in assessing the functional or hemodynamic relevance of detected lesions. To address this gap, CTP can be utilized as a complementary technique, enabling a non-invasive assessment of myocardial perfusion. Quantitative MBF measurements from dynamic CTP have shown promise for evaluating stenoses regarding their relevance to the myocardium, enhancing diagnostic accuracy when combined with CTA [2–5]. This approach may help avoiding unnecessary invasive procedures by providing clearer evidence of ischemia associated with epicardial stenosis [5].

Most applications of CTP focus exclusively on diagnosing obstructive CAD, an invasively targetable condition, in contrast to microvascular ischemia. However, microvascular ischemia is increasingly recognized as a common comorbidity and a predictor of adverse outcomes [9]; it is also considered the primary cause of ischemia in approximately 40% of selected patients without obstructive CAD [8]. Non-invasively differentiating between obstructive CAD and microvascular ischemia is challenging in a clinical routine setting, particularly when CTA findings are inconclusive. Moreover, both conditions can lead to reduced absolute and relative MBF, which restricts the accuracy of CTP, especially in patients with global or diffuse perfusion deficits, where identifying remote myocardium for comparison can be problematic.

Pathological changes such as myocardial infarction or fibrosis can alter the spatial distribution of perfusion and potentially lower the fractal dimension independently of ischemia. In our study, patients in whom delayed enhancement (as a sign of infarction or fibrosis) coincided with perfusion defects were excluded to minimize this confounding effect. In cases with small or localized infarction, fractal analysis could still be performed; however, the results preclude conclusions on the interpretability of fractal analysis in changes to the extracellular space.

Fractal analysis has been performed solely using stress CTP in this study. The addition of rest perfusion imaging might theoretically enhance interpretability in the presence of chronic infarction or fibrosis by allowing assessment of reversible versus fixed changes in fractal dimension, which warrants further investigation.

One key difference between epicardial stenosis and microvascular ischemia lies in the distinct hemodynamic effects they exert on downstream myocardial perfusion [12, 19]. In the case of epicardial stenosis, perfusion to the downstream vascular bed is affected uniformly, leading to a more homogeneous reduction in sub-endocardial vasodilator capacity across the impaired myocardial region (with a transmural gradient), and hence, a more homogeneous pattern of perfusion reduction. In contrast, microvascular ischemia is thought to affect myocardial tissue in a patchy or diffuse pattern, with diverse mechanisms contributing to a heterogeneous exhaustion of vasodilator capacity (not necessarily with a pronounced transmural gradient). These mechanisms may include endothelial dysfunction, microvascular constriction, luminal inward remodeling, and other structural or functional abnormalities in the small vessels, leading to a more complex, irregular distribution of perfusion reduction within the myocardial tissue [19, 20].

Measurements of MBF, whether absolute or relative, do not capture these perfusion patterns because they only provide a scalar measure of blood flow without reflecting its spatio-temporal distribution. Fractal analysis, in contrast, offers a quantitative assessment of these differences, allowing for indirect conclusions on the scale-invariant, fractal geometry of the underlying vascular tree [11, 21]. By calculating the fractal dimension of the perfusion pattern, fractal analysis can quantitatively characterize its complexity, therefore distinguishing between the more homogeneous vascular impairment of epicardial stenosis and the more complex pattern of microvascular ischemia.

Fractal analysis is well suited to cases of diffuse myocardial ischemia or globally reduced flow, as it quantifies the local spatio-temporal complexity of myocardial perfusion and does not depend on identifying presumably normal remote myocardium for comparison. This means it remains applicable when perfusion is globally reduced or when ischemia is diffuse, conditions in which MBF measurements (absolute and relative) can be unreliable. In such cases, fractal analysis can still distinguish perfusion patterns typical of obstructive CAD (characterized by strong reduction in fractal dimension) from those of microvascular disease (moderate reduction in fractal dimension), as shown in the low remote group in our study.

In terms of clinical workflow, fractal analysis can be implemented into standard CTA and CTP protocols as a post-processing tool, similar to how MBF maps are currently integrated. Fractal analysis provides an additional quantitative marker, which is particularly valuable in patients with inconclusive findings or overlapping epicardial and microvascular pathology [11]. This may help to assess the hemodynamic relevance of intermediate stenoses to the myocardial vascular bed. Hence, the fractal dimension might be a criterion to inform decisions about further invasive testing and to reduce false-positive results for obstructive CAD in patients with global perfusion reduction. Integrating fractal analysis into the diagnostic process could, therefore, improve patient selection for revascularization versus medical management.

Criteria established by the Coronary Vasomotion Disorders International Study (COVADIS) Group provide a comprehensive clinical diagnostic framework for microvascular angina in patients who present with ischemic symptoms and lack significant obstructive CAD (stenosis < 50%, FFR > 0.8) on coronary angiography either using CTA or ICA [22]. Evidence of impaired microvascular function constitutes a key element of COVADIS criteria and is often determined through invasively measuring the index of microcirculatory resistance (IMR) or coronary flow reserve (CFR). However, the diagnosis requires extensive, mostly invasive testing, since non-invasive alternatives to testing the CFR criterion, such as positron-emission tomography using freely diffusible tracers (e.g., ^15^O-water), are not widely available. Here, fractal analysis holds promise as an alternative non-invasive method using standard CT imaging equipment, which can be integrated into clinical routine. Its ability to accurately identify microvascular dysfunction even in the presence of epicardial stenosis might improve non-invasive characterization of patients with chronic myocardial ischemia.

This study has limitations: first, this observational study reports on an Eastern Asian multi-center population with suspected or known CAD referred for ICA. Therefore, prospective validation in larger, more diverse cohorts is warranted to confirm our findings. Second, ^15^O-water PET was not available as reference standard for validating MBF measurements and estimating myocardial perfusion reserve. Third, we excluded nine patients in whom perfusion defects coincided entirely with the presence of delayed enhancement. In such territories, interpretation of fractal analysis remains uncertain due to potential remodeling effects associated with infarction or fibrosis, which might alter perfusion complexity and the fractal dimension independently of inducible ischemia (e.g., via collateral-related microvascular changes or alterations of the extracellular space). In addition, patients with prior coronary artery bypass grafting (CABG) were excluded, which may have further reduced the heterogeneity of the study population. Fourth, supplementing routine CTA with dynamic CTP increases radiation exposure, which is a factor to consider, albeit our reported dose is comparable to that in similar studies. Fifth, all imaging in this study was performed on CT systems from a single vendor, and reproducibility of MBF and FD values across different CT platforms could not be assessed. Finally, while fractal analysis accurately characterized ischemia in our study’s population sample, more research is needed to determine whether fractal metrics correlate with clinical outcomes, as well as to investigate their utility in monitoring treatment response (e.g., after revascularization or after initiation of optimal medical therapy).

In conclusion, fractal analysis of dynamic stress CTP significantly improves the diagnostic accuracy for detecting obstructive CAD, particularly in patients with reduced remote flow and microvascular ischemia components. By characterizing the spatial complexity of myocardial perfusion, fractal analysis addresses an unmet clinical need in the non-invasive diagnosis of microvascular ischemia, especially in patients with intermediate epicardial stenosis. Hence, fractal analysis may help guiding clinical decision-making in patients with chronic myocardial ischemia. Prospective research is warranted to validate these findings and explore the potential clinical impact of integrating fractal analysis into ischemia assessment workflows.
